# A Blueberry-Enriched Diet Improves Renal Function and Reduces Oxidative Stress in Metabolic Syndrome Animals: Potential Mechanism of TLR4-MAPK Signaling Pathway

**DOI:** 10.1371/journal.pone.0111976

**Published:** 2014-11-05

**Authors:** Anand R. Nair, Carrie M. Elks, Jorge Vila, Fabio Del Piero, Daniel B. Paulsen, Joseph Francis

**Affiliations:** 1 Comparative Biomedical Sciences, School of Veterinary Medicine, Louisiana State University, Baton Rouge, Louisiana, United States of America; 2 Veterinary Clinical Sciences, School of Veterinary Medicine, Louisiana State University, Baton Rouge, Louisiana, United States of America; 3 Pathobiological Sciences, School of Veterinary Medicine, Louisiana State University, Baton Rouge, Louisiana, United States of America; 4 Adipocyte Biology Laboratory, Pennington Biomedical Research Center, Baton Rouge, Louisiana, United States of America; Universidade de São Paulo, Brazil

## Abstract

**Background:**

Metabolic syndrome (MetS) is characterized by a cluster of health factors that indicate a higher risk for cardio-renal diseases. Recent evidence indicates that antioxidants from berries are alternative to attenuate oxidative stress and inflammation. We tested the hypothesis that inflammation-induced renal damage is triggered by the activation of TLR4, and subsequent modulation of redox-sensitive molecules and mitogen-activated protein kinase (MAPK) pathway.

**Methods:**

Five-week old lean and obese Zucker rats (LZR and OZR) were fed a blueberry-enriched diet or an isocaloric control diet for 15 weeks. A glucose tolerance test and acute renal clearance experiments were performed. Gene and protein expression levels for TLR4, cytokines and phosphorylation of ERK and p38MAPK were measured. Kidney redox status and urinary albumin levels were quantified. Renal pathology was evaluated histologically.

**Results:**

Control OZR exhibited lower glucose tolerance; exacerbated renal function parameters; increased oxidative stress. Gene and protein expression levels of TLR4 were higher and this was accompanied by increased renal pathology with extensive albuminuria and deterioration in antioxidant levels in OZR. In addition, OZR had increased phosphorylation of ERK and p38MAPK. Blueberry-fed OZR exhibited significant improvements in all these parameters compared to OZR.

**Conclusion:**

TLR4-MAPK signaling pathway is a key to the renal structural injury and dysfunction in MetS and blueberry (BB) protect against this damage by inhibiting TLR4.

**Significance:**

This is the first study to put forth a potential mechanism of TLR4-induced kidney damage in a model of MetS and to elucidate a downstream mechanism by which blueberry exert their reno-protective effects.

## Introduction

Metabolic syndrome (MetS) is characterized by a cluster of health factors that indicate a higher risk for renal dysfunction and cardiac diseases. The prevalence of MetS in the United States is increasing at an alarming pace, 34% of adults being affected as of 2006 [1]. The common factors that contribute to the development of MetS include obesity, diabetes, hypertension, and hyperglycemia. MetS develops as a result of an imbalance among dietary intake, sedentary lifestyle, glucose metabolism and cardiac control [2]. Interestingly, all of these factors are also play a crucial role in proper functioning of the kidneys. Oxidative stress triggered by the overproduction of reactive oxygen species (ROS) or inefficient antioxidant systems is also involved in the development of renal injury [3]. A wide range of pharmacological therapeutics is available to combat the factors that lead to MetS, but most have side effects. In this context, the need for non-pharmacological approaches to delay the progression of MetS is growing. Many fruits, especially berries, have been shown to be excellent sources of antioxidant compounds, such as anthocyanins and phenolics [3].

Blueberries (BB), (*Vaccinium sp.)* have the highest antioxidant capacity among fruits, supplemented by anthocyanins, proanthocyanins, flavanols and phenolic acid present in them [4]. BB possess known anti-inflammatory and antioxidant properties. We have previously shown that hypertensive rats have increased production of proinflammatory cytokines (PIC) and renin-angiotensin system (RAS) components [3] and when fed with a BB diet, exhibit reduced oxidative stress and improved nephropathy [5]. We recently established that BB protect against LPS-induced acute kidney injury by modulating TLR4 expression [6]. Although previous studies have reported the beneficial effects of BB, the mechanism by which BB antioxidants protect against MetS-induced renal injury has not been explored.

Inflammation triggered by excessive PIC production is associated with hypertension-induced renal injury and cardiac pathology [7]. PIC have been shown to exacerbate ROS generation [8], [9], which can activate several intracellular signaling pathways including the NFκB pathway [3]. The NFκB signaling pathway leads to the production of more PIC, which in turn increases ROS generation and therefore gives rise to a vicious cycle. In MetS, at the cellular level, when a cell is under stress from increased fat or sugar concentration, the cell surface receptors detect the changes in the external environment, trigger the production of PIC, and thereby generate ROS. Hence, the cell surface receptors play an important role in initiating inflammation in MetS.

Toll-like receptors (TLRs) are pattern recognition receptors that play key roles in the innate immune system. TLRs detect pathogens or danger-associated molecular patterns and initiate immune cell responses. Toll-like receptor 4 (TLR4), in particular, is shown to induce cytokine production and expression of co-stimulatory molecules [10] via the NFκB signaling pathway [11], [12]. TLR4 expression has been increased in human macrophages in lipid-rich plaques [13], indicating TLR4 activation by hyperlipidemia. Further, TLR4, along with TLR2, has been shown to be increased in adolescents with MetS [14].

Mitogen-activated protein kinases (MAPKs) are important mediators of inflammation-induced tissue injury. Interestingly, MAPKs are critical downstream regulators of TLR pathways [15], [16] Although MAPK activation has been studied in inflammation-associated organ dysfunction [17], [18] and has been shown to contribute to insulin resistance [19], the involvement of TLR4-mediated MAPK phosphorylation in MetS-induced kidney dysfunction has been poorly understood. The objective of this study was to assess the role of TLR4 in MetS-induced renal damage and to understand the mechanisms by which BB protect against CKD. We hypothesized that the renal damage induced by MetS is caused by TLR4 activation and that BB protect against the damage by inhibiting TLR4 expression and subsequent downstream MAPK phosphorylation.

## Materials and Methods

### Ethics Statement

All experimental procedures were in compliance with all applicable principles set forth in the National Institutes of Health 2011 Guide for the Care and Use of Laboratory Animals. This study was approved by the Institutional Animal Care and Use Committee of the Louisiana State University School of Veterinary Medicine (protocol approval number 09-008).

### Animals

Five-week old heterozygous LZR (*fa/+*) and homozygous OZR (*fa/fa*) were purchased from Harlan and housed in a temperature- (23±2°C), humidity- and light- (12 hour light/dark cycle) controlled environment. The baseline body weights of the rats ranged from 130 to 150 grams.

### Experimental design

Rats were randomly divided into four different groups: LZR Control (LZRCC), LZR Blueberry (LZRBB), OZR Control (OZRCC), and OZR Blueberry (OZRBB). Animals were fed control or BB-enriched diets for 15 weeks. All animals were subjected to acute determination of glomerular filtration rate (GFR) and renal plasma flow (RPF) at the end of the 15 week feeding period. Rats were euthanized and kidneys were excised for analyses. Kidneys were formalin-fixed, paraffin-embedded, and then sectioned (3 um).

### Diets

Diets were prepared by Harlan Teklad (Madison, WI) using a reformulated NIH-31 diet by adding 20 g/kg lyophilized BB or 20 g/kg dried corn. The 2% BB diet was prepared by homogenizing the berries in water, lyophilizing and adding the preparation to the NIH-31 rodent chow. The control diet was prepared with corn instead of BB and the amount of corn in the control diet was adjusted to compensate for the added volume of BB, in order to make the two diets isocaloric [5]. The food consumption was measured weekly for all four groups.

### Glucose tolerance test

Blood was obtained by tail nick in conscious, restrained animals. Glucose was measured with a handheld glucose meter (One Touch Ultra, USA). Baseline fasting blood glucose was determined after an overnight fast. A glucose bolus (2 g/kg of body weight) was then administered intraperitoneally, and blood glucose was measured at 15, 30, 45, 60, 90, 120, 150, and 180-minute intervals.

### Acute renal clearance experiments

The OZR animals have been shown to exhibit reduced renal function parameters, namely glomerular filtration rate (GFR) and renal bloof flow (RBF) even at 12–14 weeks of age. [20], [21] The average GFR of male obese zucker rat at 20 weeks of age was 0.3 ml/min/g kidney wt.[21] and RBF at 14 weeks were shown to be 6 ml/min/g kidney wt Rats from all the study groups were subjected to renal clearance experiments at the end of the feeding period [3], [6]. Briefly, each rat was anesthetized with Inactin (thiobutabarbital; 100 mg/kg body weight), the right inguinal area was shaved, a small incision made, and femoral vessels were carefully isolated. The femoral artery was cannulated with heparin-primed (100 U/ml) PE-50 polyethylene tubing connected to a pressure transducer (PowerLab data acquisition systems; AD Instruments, Colorado Springs, CO) for continuous measurement of arterial pressure. The femoral vein was catheterized with heparin-primed PE-50 tubing for infusion of solutions at 20 µl/min. An isotonic saline solution containing 6% albumin was infused during surgery. After surgery, the infusion solution was replaced with isotonic saline containing 2% bovine serum albumin (BSA), 7.5% inulin (Inutest) and 1.5% PAH, and a 300 µl bolus of this solution was administered at the start of each clearance experiment. The bladder was exposed via a suprapubic incision and catheterized with a PE-200 tube for gravimetric urine collection. After a 15–20 minute stabilization period, a 30 minute clearance period was conducted to assess values of renal hemodynamic parameters. An arterial blood sample was collected at the end of the 30 minute clearance collection period for measurement of plasma inulin and PAH concentrations as previously described ([5], [6]. Plasma inulin and PAH concentrations were measured colorimetrically to determine GFR and RPF, respectively.

### Glomerular injury scoring

A semi-quantitative glomerular scoring method was used, based upon previously published methods for glomerular scoring [5]. The method was expanded to include parietal metaplasia of Bowman’s capsule, glomerular sclerosis, proteinuria and interstitial nephritis.

### Measurement of Urinary Albumin levels

Urine albumin was assessed in animals from all experimental groups using the NephratII Albumin Assay Kit (Exocell Inc., Philadelphia, USA) according to manufacturer’s instructions.

### Electron paramagnetic resonance (EPR) spectroscopy

Total ROS, superoxide, and peroxynitrite production rates were measured in pieces of kidney cortex via EPR spectroscopy as previously described [5], [6], [22]–[25]. The term ‘total ROS’ represents all ROS; however, the major sources trapped by the spin trap used are superoxide, hydrogen peroxide, and hydroxyl radical, with other species as minimal contributors. Briefly, tissue pieces were incubated at 37°C with 6.6 µl of CMH (200 µm) for total ROS measurement; 1.5 µl of PEG-SOD (50 U/µl) for 30 minutes, then CMH for an additional 30 minutes for superoxide measurement; or 30 µl of CPH (500 µm) for 30 minutes for peroxynitrite measurement. Aliquots of incubated probe media were taken in 50 µl disposable glass capillary tubes (Noxygen Science Transfer and Diagnostics, Elzach, Germany) for determination of total ROS, superoxide or peroxynitrite production, under previously established EPR settings [3], [5], [6].

### RNA extraction and Real-Time PCR

Real-time PCR was used to determine the expression levels of kidney cortex (KC) PIC and TLR4. Total RNA was isolated using Trizol reagent (Invitrogen, CA). The RNA concentration was calculated from the absorbance at 260 nm and RNA quality was assured by the 260/280 ratio. The RNA samples were treated with DNase I (Ambion) to remove any genomic DNA. First strand cDNA were synthesized from 2 µg RNA with iScript cDNA synthesis kit (Bio-rad, Hercules, CA). Real-Time PCR was performed in 384-well PCR plates using iTaq SYBR Green Super mix with ROX (Bio-rad) in triplicate using the ABI Prism 7900 sequence detection system (Applied Biosystems, Foster City, CA). The PCR cycling conditions were as follows: 50°C for 2 mins, 95°C for 3 min, followed for 45 cycles (15 s at 95°C and 1 min at 60°C). To confirm the specific PCR product, a dissociation step (15 s at 95°C, 15 s at 60°C and 15 s at 95°C) was added to check the melting temperature. Gene expression was measured by the Δ ΔCT method and was normalized to 18 s RNA mRNA levels. The data presented are the fold changes of the gene of interest relative to that of the control animals.

### Western Blotting analysis

Primary rat TLR4 (sc-30002), IL-1β (sc-7884), IL-18 (sc-7954), Nrf2 (sc-722), Keap1 (sc-33569) antibodies were purchased from Santa Cruz Biotechnology, USA and p38MAPK (#9212), p-p38MAPK (#9211), ERK1/2 (#9102), p-ERK1/2 (#9106) from Cell Signaling, USA and used following manufacturer-recommended dilutions, followed by a horseradish peroxidase-conjugated anti-rabbit IgG antibody (SC-2004, Santa Cruz Biotechnology, Inc). Rat primary anti-GAPDH (sc-25778) or anti-actin (sc-1616) was used to confirm the loading and the transfer. We used ImageJ software to analyze the bands.

### Measurement of cortical catalase and superoxide dismutase levels

Antioxidant levels in renal cortex of animals from all experimental groups were quantified. Catalase levels were assessed with a catalase assay kit (Cayman Chemicals, Ann Harbor, MI) and superoxide dismutase enzyme levels were determined using a SOD assay kit (Dojindo Molecular Technologies, Rockville, Maryland) according to manufacturer’s instructions.

### Quantification of NFκB p65 activity

The NFκB/p65 activity ELISA (Active Motif, USA) kit was used to assess the binding activity of free NFκB p65 in nuclear extracts, as described previously [6]. A sandwich ELISA method was employed to perform the analysis, according to the manufacturer’s instructions.

## Results

### Body weight and food intake

Food consumption and body weight were measured weekly. Food intake did not differ between LZRCC and LZRBB animals or between OZRCC and OZRBB groups. The initial and final body weights of the animals in all groups are summarized in Table 1. OZRCC and OZRBB had significantly higher body weights compared to the LZR animals that were fed either diet. There was no significant difference between the body weights of OZR rats, irrespective of the diet.

**Table 1:** Average initial and final body weight of rats.

Body Weight	LZR		OZR	
	CC	BB	CC	BB
Initial weight (g)	**158.4±3.99**	**163.9±2.96**	184.2±3.56*	181.6±5.14*
Final weight (g)	**554±12.07**	**531.7±15.26**	840.1±42.64*	814±46.24*

The body weight of all rats was measured at the start and end of the feeding period. All values are presented as mean ± SEM (*-p<0.05 vs. LZRCC, ^#^-p<0.05 vs. OZRCC, ^$^-p<0.05 vs. OZRBB).

### BB-enriched diet improves glucose tolerance in MetS animals

**Figure 1:**
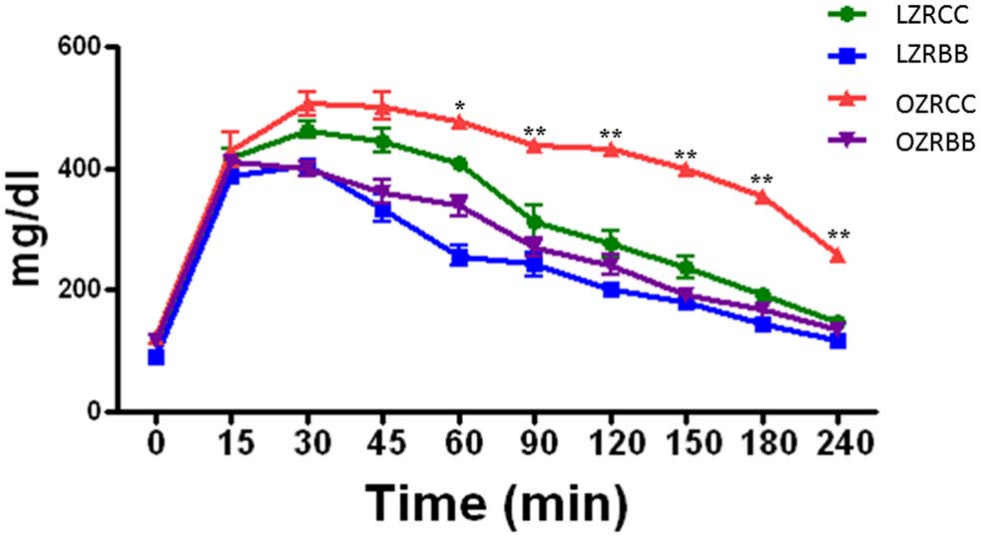
Effect of BB feeding on glucose sensitivity in MetS animals. The glucose sensitivity of rats from all experimental groups as assessed by glucose sensitivity assay (n = 7 per group). All values are presented as mean ± SEM (*p<0.05, **p<0.01).

The control OZR had impaired glucose tolerance compared to the LZR. The improvements in glucose tolerance over different time-points in BB-fed OZR rats appear in Figure 1. BB feeding for a period of 15 weeks improved glucose tolerance in OZRBB rats compared to the OZRCC group. However, there was no significant difference in the glucose sensitivity between LZRCC and LZRBB rats.

### Kidney function is impaired in OZR and blueberry improves renal hemodynamic parameters in these animals

**Figure 2:**
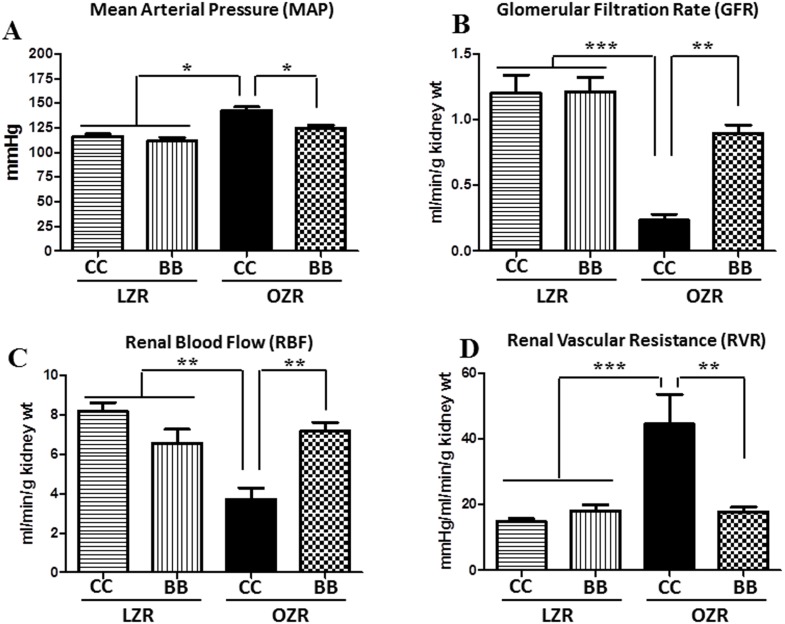
Effect of BB on renal hemodynamic dysfunctions in MetS animals. The kidney function of rats from all experimental groups as assessed by renal clearance experiments (n = 6–9 per group) (A) BB supplementation significantly decreased the MAP in obese zucker rats (B) MetS animals exhibited a significantly reduced GFR. BB pretreatment improved the GFR in MetS animals. (C) MetS animals had a reduced renal blood flow. BB treatment normalized the renal blood flow in these animals. (D) The RVR was increased in MetS animals and BB prevented this increase in RVR in them. All values are presented as mean ± SEM (*p<0.05, **p<0.01, ***p<0.001).

MetS is known to induce a loss of function of the kidney, thus leading to alterations in renal hemodynamic parameters. The kidney function in rats from all study groups was assessed to determine the extent of kidney damage. The blood pressure trends for each group of rats appear in Figure 2A. The OZRCC rats had significantly higher mean arterial pressure (MAP) compared to the LZR rats. The MAP of OZRBB rats were significantly lower in comparison to OZRCC group by the end of the feeding period. Figures 2B–2D illustrates the reno-protective effects of BB-enriched diet in OZR rats fed for 15 weeks. The renal clearance experiments showed significant reductions in GFR and RBF, and an increase in renal vascular resistance (RVR) in OZRCC animals. In contrast, in the OZRBB rats, there was a significant increase in GFR and RBF, and a decrease in RVR, indicating a reno-protective effect of BB. There was no significant difference in blood pressure or renal hemodynamic measures between LZRCC and LZRBB animals.

**Figure 3:**
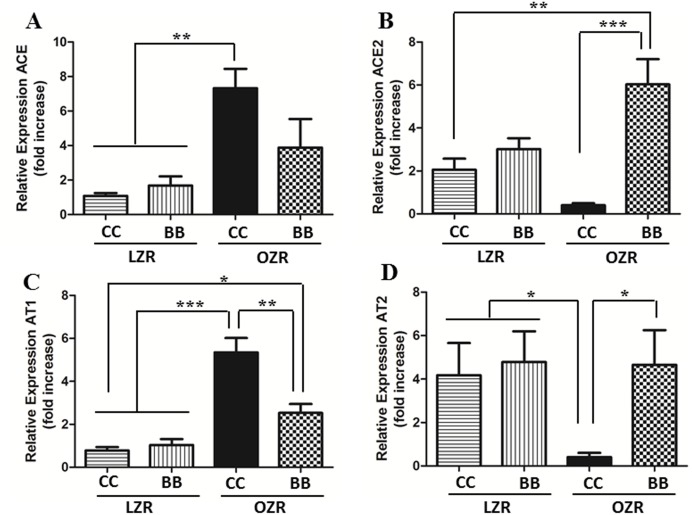
Effect of BB on renal renin-angiotensin system in MetS animals. The mRNA expression of (A) ACE, (B) ACE2, (C) AT1 and (D) AT2 in the renal cortical tissue. The gene expression of ACE and AT1 were significantly increased and ACE2 and AT2 were reduced in MetS rats. The BB enriched diet was able to attenuate these changes in renin-angiotensin system gene expression. All values are presented as mean ± SEM (*p<0.05, **p<0.01, ***p<0.001).

In addition to the kidney function parameters, we also measured the expression of renin-angiotensin system (RAS) genes in the kidneys of animals from all experimental groups (Figure 3A–3D). The OZRCC animals had an elevated expression of ACE and AT1 genes and a significantly reduced expression of ACE2 and AT2, compared to the lean controls. Conversely, BB enriched diet could significantly attenuate the expression of ACE and AT1, and also improved the expression levels of ACE2 and AT2 genes in the renal tissue of OZR rats. Taken together with the improved renal function, this result of the renin-angiotensin system components confirms a protective functional role of BB in the kidneys of MetS animals.

### Kidney structure is altered in OZR and blueberry feeding preserves glomerular morphology and structure in these animals

**Figure 4:**
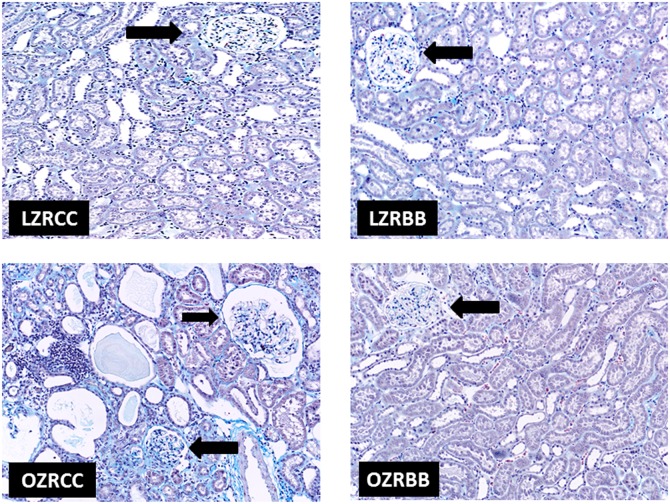
Effect of BB on renal pathology in MetS animals. (A) Representative images of the renal pathology of rats from all experimental groups as assessed by Masson’s trichrome staining of the kidney sections (n = 6). There are no significant renal structural changes visible in LZRCC and LZRBB groups. However, renal changes in the OZRCC image are evident in glomeruli, tubules and interstitium. There is moderate glomerular fibrosis; tubular dilatation and attenuation of lining epithelium with intraluminal hyaline casts; tubular atrophy; multifocal to coalescing moderate to severe interstitial fibrosis; multifocal interstitial, moderate to severe lymphocytic and plasmacytic nephritis. OZRBB animals exhibited less pathological changes with minimal interstitial fibrosis.

**Figure 5:**
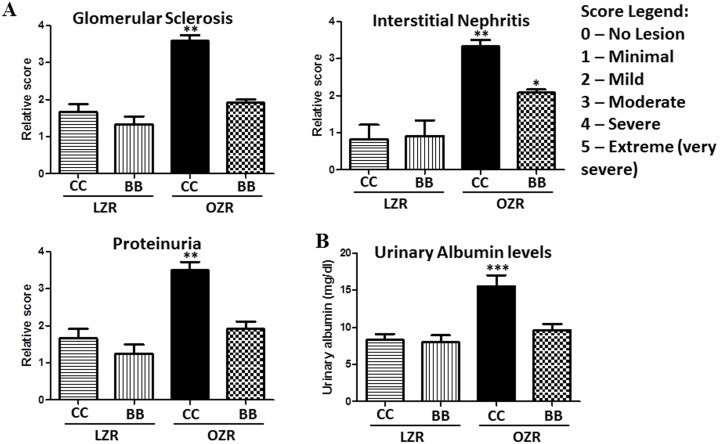
Effect of BB on renal pathology in MetS animals (pathological scoring). (A) Relative pathological scoring of glomerular sclerosis, interstitial nephritis and proteinuria as evaluated by a veterinary pathologist who was blinded to experimental conditions (n = 6). (B) Urinary albumin levels (mg/dl) in rats from all experimental groups as determined by the albumin assay kit.

Masson’s Trichrome-stained kidney sections from rats (n = 6) from all experimental groups were examined by a veterinary pathologist who was blinded to the experimental conditions. The kidneys of LZRCC and LZRBB exhibited similar appearances histologically. Representative photographs of glomeruli for each experimental group appear in Figure 4. Renal changes in the OZRCC image are evident in glomeruli, tubules and interstitium. There is moderate glomerular fibrosis; tubular dilatation and attenuation of lining epithelium with intraluminal hyaline casts; tubular atrophy; multifocal to coalescing moderate to severe interstitial fibrosis; multifocal interstitial, moderate to severe lymphocytic and plasmacytic nephritis. Remarkably, OZRBB animals exhibited less pathological changes with minimal interstitial fibrosis. When compared to OZRCC, OZRBB exhibited less severe glomerular adhesions, cortical and medullary tubular lesions, interstitial nephritis (Figure 5).

### Oxidative stress contributes to the progression of MetS while blueberry diet attenuates free radical production rates in MetS animals

Overproduction of free radicals, mainly ROS and reactive nitrogen species (RNS), is a major factor contributing to the progression of MetS. We measured total ROS, superoxide and peroxynitrite production rates using EPR spectroscopy in cortical tissues from rats (n = 7) in all experimental groups. At the kidney tissue level, the total ROS, superoxide and peroxynitrite production rates were significantly higher in OZRCC rats compared to the LZRCC and LZRBB rats. However, BB treatment attenuated the rate of cortical and medullary free radical production in the OZRBB animals. Table 2 shows the free radical production rates in cortical and medullary kidney tissues in rats from all experimental groups.

**Table 2:** Rate of generation of total ROS, superoxide and peroxynitrite in the kidney cortical and medullary tissue.

	LZR		OZR	
	CC	BB	CC	BB
**Renal Cortex**				
Total ROS (µM/mg protein/min)	**0.138±0.026**	**0.131±0.011**	0.368±0.055*^$^	0.224±0.026*^#^
Superoxide (µM/mg protein/min)	**0.048±0.009**	**0.037±0.012**	0.089±0.021*^$^	0.061±0.014*^#^
Peroxynitrite (µM/mg protein/min)	**0.017±0.005**	**0.020±0.003**	0.072±0.013*^$^	0.033±0.015*^#^
**Renal Medulla**				
Total ROS (µM/mg protein/min)	**0.074±0.012**	0.067±0.015*	0.182±0.020*^$^	0.092±0.016*^#^
Superoxide (µM/mg protein/min)	**0.027±0.008**	**0.024±0.009**	0.098±0.015*^$^	0.053±0.008*^#^
Peroxynitrite (µM/mg protein/min)	**0.011±0.006**	**0.010±0.006**	0.036±0.007*^$^	**0.016±0.010^#^**

OZRCC animals showed a significantly higher rate of total ROS, superoxide and peroxynitrite generation. Pretreatment with BB was able to inhibit the increase in the rate of free radical production. All values are presented as mean ± SEM (*-p<0.05 vs. LZRCC, ^#^-p<0.05 vs. OZRCC, ^$^-p<0.05 vs. OZRBB).

### Kidney TLR4 expression is elevated in animals with MetS and blueberry supplementation decreases TLR4 expression in the kidney

**Figure 6:**
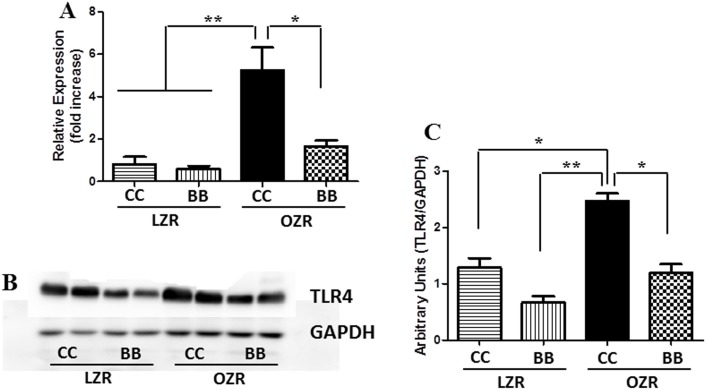
Effect of BB on renal TLR4 gene and protein expression in MetS animals. The (A) mRNA (n = 6) and (B) a representative western blot of TLR4 in the renal cortical tissues. (C) Bands were analyzed and quantified by densitometry (n = 6). The gene and protein expression of TLR4 was increased in the MetS rats. The pretreatment with BB was able to inhibit the increased expression of TLR4 in MetS animals and was comparable to the LZR controls. All values are presented as mean ± SEM (*p<0.05, **p<0.01).

TLR4 plays a vital role in activating the immune system in response to inflammation and oxidative stress. Kidney cortical TLR4 gene and protein expression was measured in tissues from all experimental groups using real time RT-PCR and western blotting technique respectively (Figure 6). Both gene and protein levels of TLR4 were elevated in OZRCC rats compared to the LZR animals, indicating that the increased inflammation in the kidney of MetS animals was, at least in part, mediated through TLR4. In contrast, OZRBB rats had an attenuated TLR4 gene and protein expression levels when compared to OZRCC rats, and comparable to LZRCC and LZRBB animals.

### MetS animals expressed increased phosphorylation of ERK and p38 MAPK and BB intervention significantly inhibited the phosphorylation of MAPK

**Figure 7:**
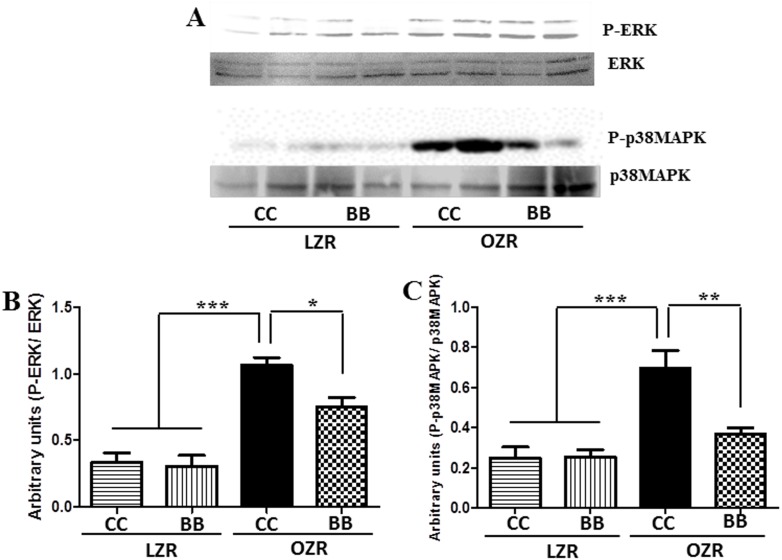
Effect of BB on MAPK signaling in MetS animals. The protein expression of total and phosphorylated MAPK (ERK and p38) was determined by western blotting. (A) A representative western blot showing ERK and p38MAPK protein expression. Western bands for ERK (B) and p38MAPK (C) were analyzed and quantified by densitometry. All values are presented as mean ± SEM (*p<0.05, **p<0.01, ***p<0.001).

We examined the expression levels of total and phosphorylated ERK and p38 in the renal cortex of animals from all experimental groups. The expression level of p-ERK and p-p38MAPK was not significantly different between the LZR animals (Figure 7A–7C). Consistent with the TLR4 expression pattern, the OZRCC exhibited an elevated expression of p-ERK and p-p38MAPK in the kidneys. In contrast, BB intervention (OZRBB) could attenuate the phosphorylation of MAPK in these animals. Taken together with the reduced TLR4 expression in MetS rats, these findings clearly demonstrate that BB pretreatment in MetS animals attenuates MAPK expression.

### NFκB activity and PIC expressions are higher in MetS and blueberry feeding attenuates these changes in MetS animals

**Figure 8:**
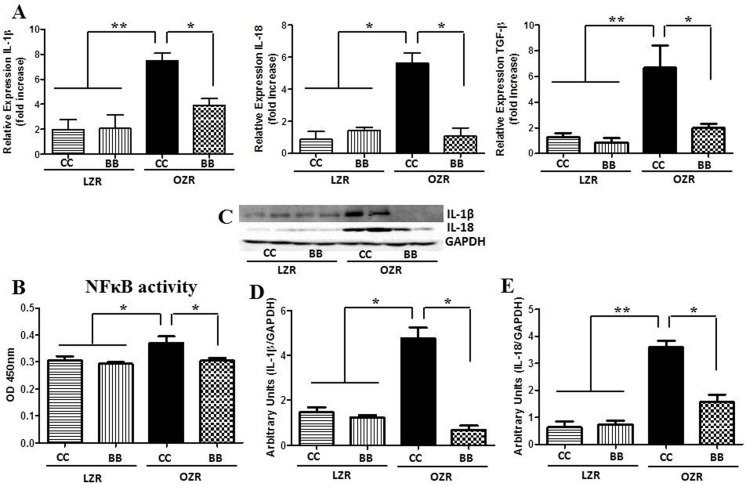
Effect of BB on the renal inflammatory profile in MetS animals. The (A) mRNA expression (n = 6) of IL-1β, IL-18 and TGF-β in the renal cortical tissues. (B) DNA binding activity of NFκB p65 subunit in renal cortical tissues of rats from each experimental group (n = 6), as determined by ELISA. Increased NFκB p65 DNA binding activity in MetS animals was significantly reduced by BB treatment. (C) A representative western blot showing IL-1β and IL-18 protein expression. Western blot bands for IL-1β (D) and IL-18 (E) were analyzed and quantified by densitometry. All values are presented as mean ± SEM (*p<0.05, **p<0.01).

The activation of TLR4 was confirmed by measuring the NFκB activity. We measured the renal cortical NFκB p65 DNA binding activity in tissues from control and BB treated groups (n = 6). Consistent with the TLR4 expression results, rats from OZRCC group had significantly higher cortical NFκB activity compared to LZRCC and LZRBB groups (Figure 8B). The OZRBB rats had a significantly lower NFκB activity levels compared to the OZRCC rats suggesting that the BB supplementation prevents the increase in NFκB activity seen in MetS animals.

Gene expression levels of proinflammatory cytokines IL-1β, IL-18, TNFα and protein expression levels of IL-1β and IL-18 were measured in all experimental groups (n = 6). These interleukin levels were significantly reduced in the OZRBB compared to the OZRCC animals (Figure 8A, 8C–8E, Figure S1). Again, there was no significant difference in the expression of these proteins between the two groups of LZR animals. The difference in the expression of IL-1β and IL-18 between the OZR groups is particularly important not only because these are proinflammatory molecules, but also as IL-1β and IL-18 are downstream molecules of the TLR4 pathway. TNFα mRNA expression results were also consistent with the expression of other PIC.

### MetS animals had a reduction in antioxidant defense which was restored by the blueberry diet

**Figure 9:**
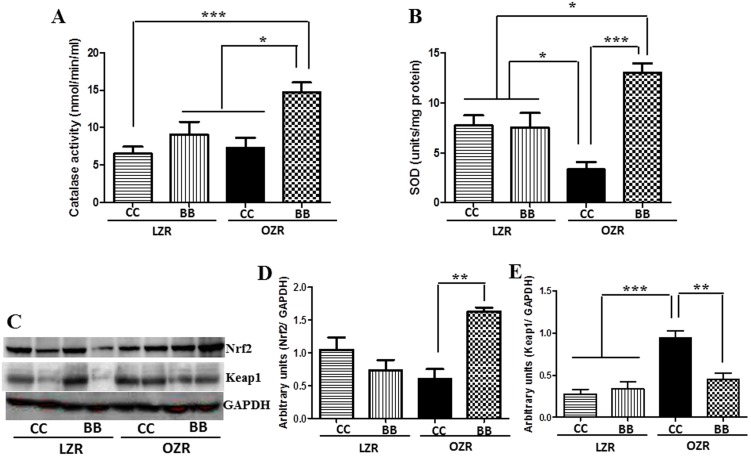
Effects of BB on antioxidant enzymes and Nrf2-Keap1 signaling in MetS animals. Renal cortical catalase (A) and SOD (B) levels as determined by commercially available kits (n = 6). (C) A representative western blot showing Nrf2 and Keap 1 protein expression. Western bands for Nrf2 (D) and Keap1 (E) were analyzed and quantified by densitometry. All values are presented as mean ± SEM (*p<0.05, **p<0.01, ***p<0.001).

We measured the cortical levels of the antioxidant enzymes SOD and catalase in all experimental groups (Figure 9A–9B). SOD catalyzes the conversion of superoxide into oxygen and hydrogen peroxide. Catalase breaks down hydrogen peroxide. The SOD and catalase levels were depleted in rats belonging to the OZRCC group compared to the LZR groups. A blueberry-enriched diet significantly increased the SOD and catalase levels in the OZRBB group, indicating a beneficial antioxidant effect of BB in MetS animals.

We also determined the protein expression levels of redox sensitive transcription factor NF-E2-related factor 2 (Nrf2), which is a key regulator of antioxidant enzymes and its associated protein Kelch-like ECH associated protein 1 (Keap1) in the kidney of animals from all the groups. Nrf2 levels were significantly increased and Keap1 concomitantly reduced in the OZRBB group compared to the OZRCC (Figure 9C–9E), indicating an improved anti-oxidant defense system in BB fed MetS animals.

## Discussion

The most prominent finding of this investigation is that BB protect against chronic kidney disease in the Zucker rat model of metabolic syndrome by attenuating TLR4 expression and reducing oxidative stress in the kidney. To the best of our knowledge, this study is the first to demonstrate that 1) TLR4 signaling and consequent inflammatory cascade, at least in part, mediates the renal dysfunction in MetS animals; 2) TLR4-mediated phosphorylation of ERK and p38MAPK could be a possible mechanism for the progression of chronic kidney injury in MetS; 3) BB can protect against chronic kidney injury in MetS animals by inhibiting TLR4 and subsequently attenuating MAPK activity; and 4) BB can improve other contributing factors to MetS, such as glucose metabolism, by reducing inflammation and improving the redox balance. The ability of BB to inhibit TLR4 could be an important advancement towards the identification of novel therapeutic strategies.

MetS is a progressive health disorder that predisposes an individual to cardio-renal dysfunction [26] and its increasing incidence is a matter of concern. The most effective way to prevent kidney disease induced by MetS is to prevent the incidence of MetS or to delay its progression. However, the mechanism by which MetS exacerbates kidney disease remains elusive. In this study, we used zucker rats which are established genetic models of research for MetS. The obese zucker rats (recessive) are characterized by obesity, hyperinsulinemia, hypertension and hyperlipidemia. Interestingly, inflammation and oxidative stress have been implicated in all these pathological conditions and also in CKD [27]. Therefore, we sought to investigate the mechanism that regulates the CKD in these animal models of MetS. We also studied if a preventive intervention by BB could attenuate the structural and functional damage to the kidney in these MetS animals. Further, we tried to elucidate a molecular mechanism by which BB protects against MetS-induced kidney dysfunction.

Chronic kidney disease is characterized by a loss of function of the kidneys, which disrupts the body fluid homeostasis. Recent studies have demonstrated that kidney disorders are impending in MetS [27] and TLR4 blockade protects kidney in type-2 diabetic mice via modulating the inflammatory signaling [28]. In this study, we analyzed renal functional parameters in all experimental groups. Our findings indicate that the OZRCC rats had a marked decrease in kidney function as evidenced by a reduced GFR and RBF, and increased RVR when compared to the lean controls. These findings are consistent with previous studies demonstrating that MetS and its components are associated with a reduction in GFR [29], [30]. However, renal functional parameters were normalized in the blueberry fed OZR (OZRBB) rats, demonstrating a significant increase in the GFR and RBF, and decrease in the RVR compared to their control diet-fed obese counterparts.

Abnormal activation of the RAS is an important mediator of kidney damage [31] and has proinflammatory effects that in turn affect the renal hemodynamic parameters [32]. We measured the gene expression levels of RAS components (ACE, ACE2, AT1, AT2) to determine their possible role in the renoprotective effect exerted by BB in this study. We found a downregulation of the genes (ACE and AT1) in the vasoconstrictor arm of the RAS, and a concomitant upregulated expression of genes belonging to the vasodilatory arm of RAS, namely ACE2 and AT2, in the kidney of BB-fed OZR rats, when compared to the control OZR rats. These findings suggest that BB-exerted reno-protective effect can be attributed partly to improved RAS components in the kidney and indicates yet another pleiotropic effect of BB.

Oxidative stress induced generation of reactive oxygen species (ROS) and other free radicals have been implicated in MetS [33], [34], pathogenesis of kidney diseases [35]–[37] and also in MetS-induced tubulointerstitial injury [38]. Superoxide anions have been shown to cause increased smooth muscle cell contraction by regulating cytosolic calcium concentrations [39]. Our EPR data show that the generation of ROS, superoxide and peroxynitrite were increased in the medulla and cortical tissue of the kidneys of MetS animals. This increase in the mediators of oxidative stress in the kidney tissue can induce vasoconstriction of the arterioles, thus contributing to the decreased RBF and elevated RVR in the MetS animals [40]. Further, the antioxidant defense mechanism in these OZRCC animals were weak compared to the LZRCC and LZRBB rats, as indicated by the SOD and catalase enzyme activity. The BB-enriched diet, however, was able to restore the redox balance in the MetS animals.

Obesity and hypertension are hallmarks of MetS that have been implicated in kidney diseases. In addition to the injury to the kidney tissue [41], proteinuria and microalbuminuria have been linked to MetS-associated CKD [27], [42], [43]. Studies have also shown that glomerulopathy [41] and injury-induced reduction in the absorption by the tubules result in proteinuria [44]. Our histopathological data clearly indicates a marked disruption of the renal structure in the OZRCC animals compared to the LZR rats. This structural damage in OZRCC animals included glomerular sclerosis, interstitial nephritis, fibrosis and proteinuria. We also examined the gene expression of TGF-β, which is an important profibrotic marker [45]. The OZRCC animals had an elevated expression of TGF-β (compared to LZRCC and LZRBB) which was significantly reduced in OZRBB group. Interestingly, previous studies have correlated renal TLR4 expression with the inflammatory marker TGF-β in CKD [46]. An increase in TGF-β mRNA levels has been implicated in the glomeruli of diabetic rats [47]. Further, we also measured the albuminuria levels in these animals and our results were consistent with the histopathological findings. BB was able to exert a reno-protective effect by attenuating nephropathy and albuminuria in OZRBB rats.

Inflammation is one of the major contributors to the progression of MetS-induced kidney disorders [48], [49]. TLR4 signaling pathways and their expression have been studied in relation to inflammation in kidney injury [6], [50]. We examined the gene and protein expression of TLR4 in the kidney cortical tissues of animals from all experimental groups. Interestingly, the gene and protein expression of TLR4 was significantly increased in the OZRCC rats compared to the LZRCC and LZRBB groups. The TLR4 signaling-driven inflammation is, at least in part, a possible cause for the progression of glomerular and tubular injury in the MetS animals, thereby contributing to renal dysfunction. The MetS animals that were fed on a BB-diet had a reduced gene and protein expression of TLR4 in the kidney cortex, thus indicating a protective role of BB in the kidney of these animals.

Further, to elucidate the downstream mechanism of reno-protection by BB, we examined the association of TLR4-mediated MAPK activation in MetS-induced kidney dysfunction. Previous findings have established a potential link between ERK1/2 phosphorylation and oxidative stress-induced insulin resistance [19], which is a characteristic of MetS. Xi L. et al recently showed that selective inhibition of ERK1/2 in rat hepatocytes improved an impaired insulin signaling [51]. In addition, inhibition of p38MAPK signaling pathways have been shown to prevent diet-induced MetS in rats [52]. Although MAPK activation has been shown in LPS-induced kidney dysfunction [17], [18], [53], a role for the TLR4-MAPK pathway in MetS-induced CKD has never been investigated. Our findings demonstrate phosphorylated-ERK and p38MAPK in OZRCC animals compared to the LZRCC and LZRBB. Interestingly, BB-treated OZR -had suppressed ERK and p38MAPK activity in the kidneys. This inhibition of MAPK activation in OZRBB animals could also be a plausible explanation for the improved glucose sensitivity in these animals. Therefore, these results not only designate a key role of TLR4-MAPK signaling in MetS-associated CKD but also indicate a potential mechanism by which BB protects against chronic kidney injury.

In addition to attenuated TLR4 and MAPK expression, OZRBB exhibited decreased expression of PICs. We examined the expression of gene and protein expression of IL-1β and IL-18 in the kidney cortex of animals from all experimental groups. The importance of IL-1β and IL-18 expression patterns in this study is particularly important, not only because these are proinflammatory molecules, but also as these are downstream molecules of the TLR4 pathway. We observed that OZRBB animals expressed a significantly lower amount of IL-1β and IL-18 compared to the OZRCC rats. This is consistent with the previous findings from our lab, which reported that in SHR, a renal function decline involves an association of PICs with their transcription factor NFκB [3]. Further, oxidative stress is a key regulator of NFκB activity [54] and it produces a positive feedback mechanism related to inflammation and tissue injury. In this context, we also measured the NFκB activity in all the groups, as NFκB activity is an indicator of TLR4 activation. Our data shows that BB attenuates the NFκB activity in MetS rats, thus confirming the reno-protective effect of BB is, at least in part, mediated by TLR4.

NFκB and MAPK pathways regulate the expression of many genes involved in inflammation and tissue injury [55], [56] and their inhibition have been shown to have tissue protective effects. Nrf2 antioxidant pathway is a key mechanism in the attenuation of kidney injury [57] by reno-protective agents [58], [59]. Several mechanisms of Nrf2 activation have been reported, including MAPK signaling pathways. In normal physiological conditions, the MAPK family maintains the much needed balance between NFκB and Nrf2 activation [60]. In pathological conditions though, the pro-oxidative NFκB arm dominates and contributes to the increased generation of pro-inflammatory cytokines and ROS, thus weakening the anti-oxidant defense system. The Nrf2 and Keap1 protein expression were examined in the cytoplasmic extracts. Nrf2 regulates the expression of several anti-oxidant enzymes within the cell. Although Keap1 is known to regulate the cytoplasmic-nuclear shuttling of Nrf2, it also controls the degradation of Nrf2 [61]. A higher expression level of Nrf2, concomitant with a reduced expression level of Keap1, in the cytoplasmic fraction indicates increased cyto-protective activity. Therefore, we assessed the expression levels of Nrf2 and Keap1 in the cytoplasmic fraction. Our data indicate that BB intervention inhibits TLR4 in chronic kidney disease, thereby augmenting the anti-inflammatory actions of Nrf2 over the pro-inflammatory actions of NFκB. In light of these findings, we underline the anti-inflammatory and antioxidant properties of BB, wherein the reno-protective effect could be due to a suppression of inflammation and subsequent increase in anti-oxidant mechanism or vice versa.

In summary, our results suggest a major role for blueberry in protecting against MetS-associated CKD via a decrease in inflammation and most importantly, this study provides comparative evidence for the mechanism of action of BB, via inhibition of TLR4, and consequent attenuation of ERK and p38MAPK phosphorylation. This study also suggests that non-pharmacological antioxidant approaches have protective value against MetS-associated CKD and also open up the possibility of a potential target in TLR4 against renal diseases.

## Supporting Information

Figure S1 **Effects of BB on TNFα mRNA expression in the kidney cortical tissue of MetS animals.** The mRNA expression (n = 6) of TNFα in the renal cortical tissues. All values are presented as mean ± SEM (*p<0.05, **p<0.01).
(TIF)
