# Supplementary figures and images for: A Blueberry-Enriched Diet Improves Renal Function and Reduces Oxidative Stress in Metabolic Syndrome Animals: Potential Mechanism of TLR4-MAPK Signaling Pathway
Source: PLoS One. 2014 Nov 5;9(11):e111976. doi: 10.1371/journal.pone.0111976 (PMC4221362; doi:10.1371/journal.pone.0111976)

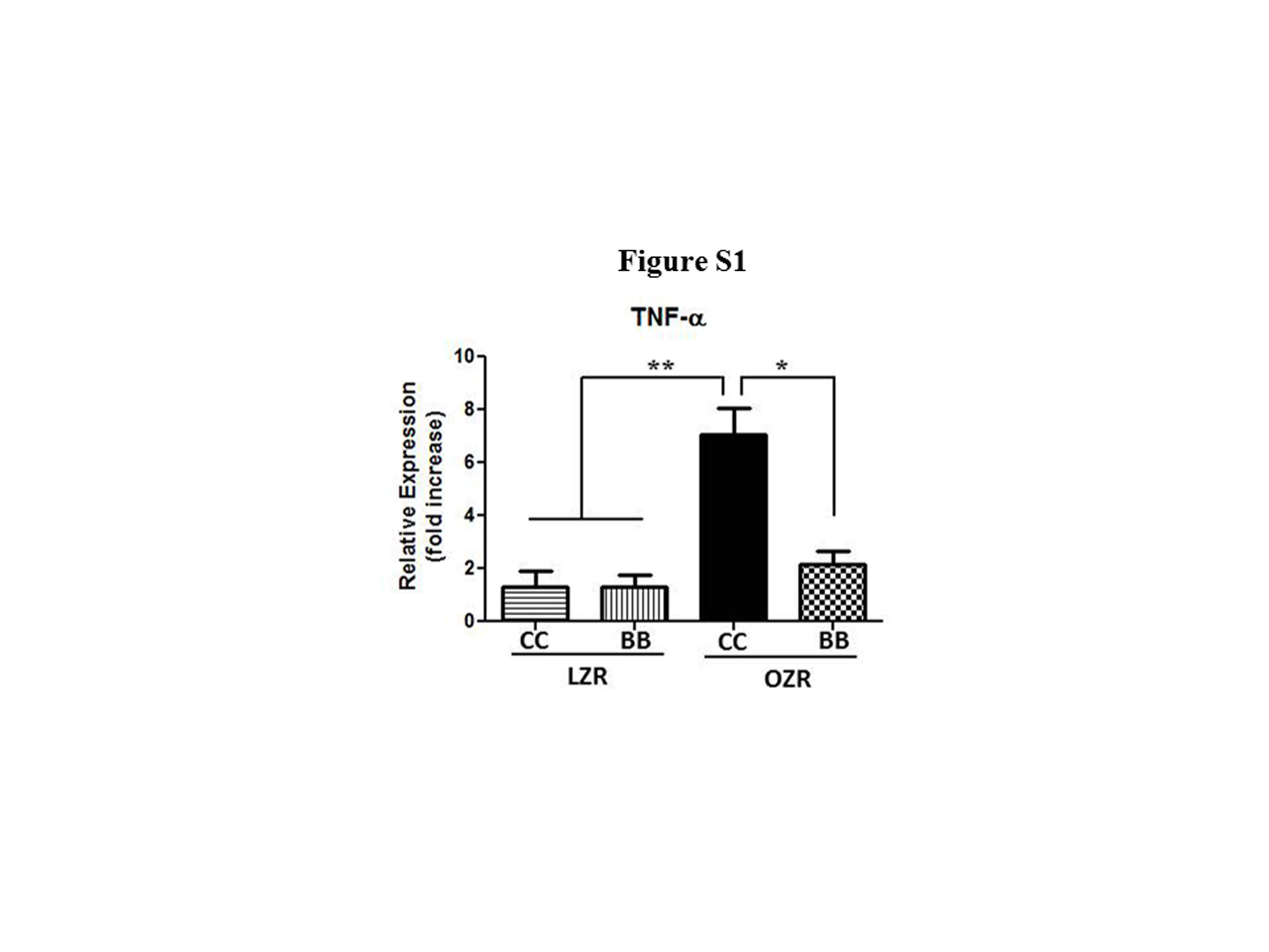

Supplement: Figure S1 — Effects of BB on TNFα mRNA expression in the kidney cortical tissue of MetS animals. The mRNA expression (n = 6) of TNFα in the renal cortical tissues. All values are presented as mean ± SEM (*p<0.05, **p<0.01). (TIF) [file pone.0111976.s001.tif]
